# Combined Neoadjuvant-Adjuvant Immunotherapy and Abdominoperineal Resection for a Primary Anorectal Melanoma: A Case Report

**DOI:** 10.7759/cureus.73931

**Published:** 2024-11-18

**Authors:** Georgios E Papanikolaou, Konstantinos Tsimogiannis, Eleftherios Kampletsas, Theocharis Chatzoglou, Dimitrios N Varvarousis

**Affiliations:** 1 Department of Plastic Surgery, Metropolitan General Hospital, Athens, GRC; 2 Department of Medicine, University of Ioannina, Ioannina, GRC; 3 Department of Colorectal Surgery, St Luke’s Hospital, Thessaloniki, GRC; 4 Department of Medical Oncology, University General Hospital of Ioannina, Ioannina, GRC; 5 Department of Orthopaedics, University General Hospital of Ioannina, Ioannina, GRC

**Keywords:** abdominoperineal resection, anorectal melanoma, immune checkpoint inhibitors, immunotherapy, mucosal melanoma

## Abstract

Anorectal melanoma (ARM) is a rare entity with aggressive biological behavior and poor prognosis. Clinically, ARM presents with atypical symptoms, such as anal pain and bleeding, thus often being misdiagnosed as a benign anorectal pathology and leading to delayed diagnosis. We present a case of a 73-year-old female patient with stage I ARM, treated successfully with a combination of neoadjuvant-adjuvant immunotherapy (nivolumab, an anti-PD-1 monoclonal antibody) and abdominoperineal resection. The patient is disease-free at a five-year follow-up after the operation, suggesting that long-term targeted immunotherapy offers a durable and promising response. This case highlights the critical role of a multidisciplinary approach, involving specialists in ARM surgery and systemic therapies, to improve prognosis and ensure an optimal quality of life for patients with ARM. Given the limited scientific evidence, further prospective and randomized trials are required to develop effective systemic therapies and improve the survival of patients with ARM.

## Introduction

Anorectal melanoma (ARM) is an uncommon and aggressive type of malignant neoplasm, accounting for less than 1.6% of all malignant melanomas, about 24% of all mucosal melanomas, and up to 4% of anorectal malignancies [1,2].

The tumor usually presents with non-specific symptoms, such as rectal bleeding, anal mass, pain, and change in bowel habits, simulating benign anorectal diseases. Moreover, these lesions are often endoscopically amelanotic, and up to 34% of tumors lack melanin histologically [2]. Therefore, the diagnostic approach to ARM can be challenging, often leading to a diagnosis at an advanced stage. Consequently, the prognosis of ARM is poor, with a five-year overall survival (OS) estimated to be 6-22%, a five-year recurrence-free survival of up to 17%, and a median survival of up to 24 months [3,4].

Given the rarity of the disease, scientific data regarding the therapeutic approach to ARM are limited. Surgical options include wide local excision (WLE) and abdominoperineal resection (APR), with no significantly different survival outcomes [5,6]. Radiotherapy and chemotherapy provide limited benefit to patients, without improving prognosis. There are only small retrospective studies and prospective trials, mainly on advanced mucosal melanoma; therefore, further research is needed to clarify the necessity of traditional therapies. Recently, the development of targeted therapies and immunotherapy has increased the survival rates of patients with mucosal melanoma, including those with ARM [6,7]. However, multimodal treatment is often necessary, such as the combination of surgery and immunotherapy. Accordingly, we present a case of a 73-year-old female diagnosed with primary ARM and successfully treated with neoadjuvant-adjuvant immunotherapy and APR.

## Case presentation

This is a case of a 73-year-old Caucasian female with a medical history of type 2 diabetes mellitus, arterial hypertension, combined hyperlipidemia, and hyperuricemia. Initially, she complained of painful defecation, bleeding per rectum, and altered bowel habits lasting for approximately three months. Therefore, she was referred to a gastroenterologist for a colonoscopy, which revealed an exophytic mass, 3 cm in length, circumferentially occupying the distal rectal lumen and infiltrating the dentate line. Multiple biopsies were taken from the lesion. The histopathologic examination was laborious and showed the presence of malignant melanoma of the rectum, while immunohistochemistry was positive for S-100 and HMB45 markers and negative for AE1/AE3, CK5/6, CK7, CK20, CEA, and p63, thereby confirming the diagnosis of mucosal melanoma.

**Figure 1:**
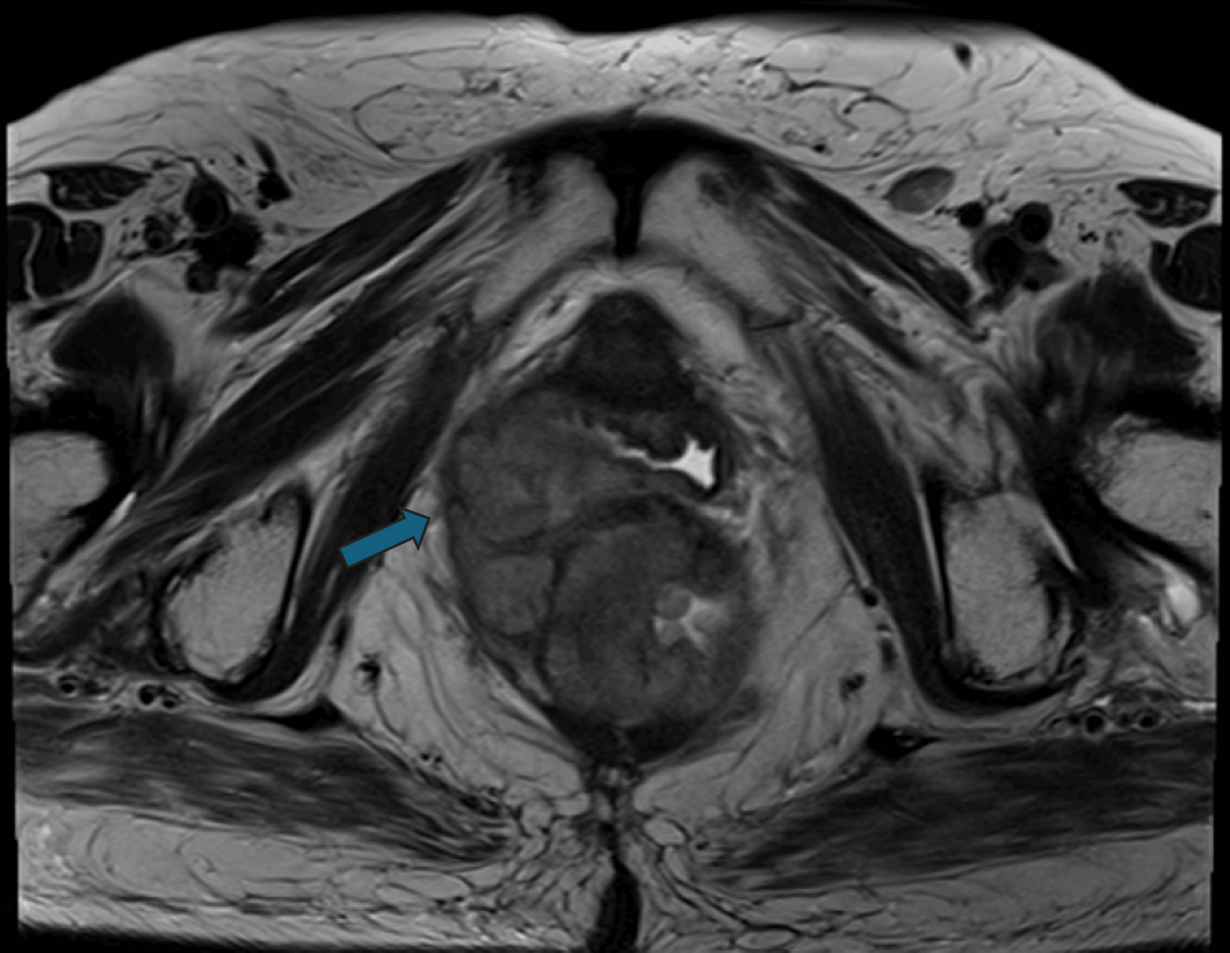
Magnetic resonance axial plane image T2 sequence showing the intraluminal rectal lesion

Consequently, the patient was referred to a different surgical team and staged with magnetic resonance imaging (MRI) of the lower pelvis. The imaging revealed the presence of a large intraluminal lesion extending from the distal rectum to the anal canal, mainly on the right side of the midline. The lesion measured 8.5 × 7.3 × 5.7 cm, with full-thickness invasion of the rectal wall and the right internal anal sphincter, as well as infiltration of the perirectal fascia and fat (Figure 1).

Additionally, fluorodeoxyglucose-positron emission tomography/computed tomography (FDG PET/CT) showed intense FDG uptake within the rectum, with no evidence of distant metastases. Genetic analysis did not identify any BRAF-V600E or c-KIT (exon 9, 11, 13, 17) mutations. Therefore, the surgical team proceeded with an exploratory laparotomy, which revealed a large, fixed rectal mass that was impossible to resect. They concluded the operation with a loop colostomy to relieve distal rectal obstruction.

**Figure 2:**
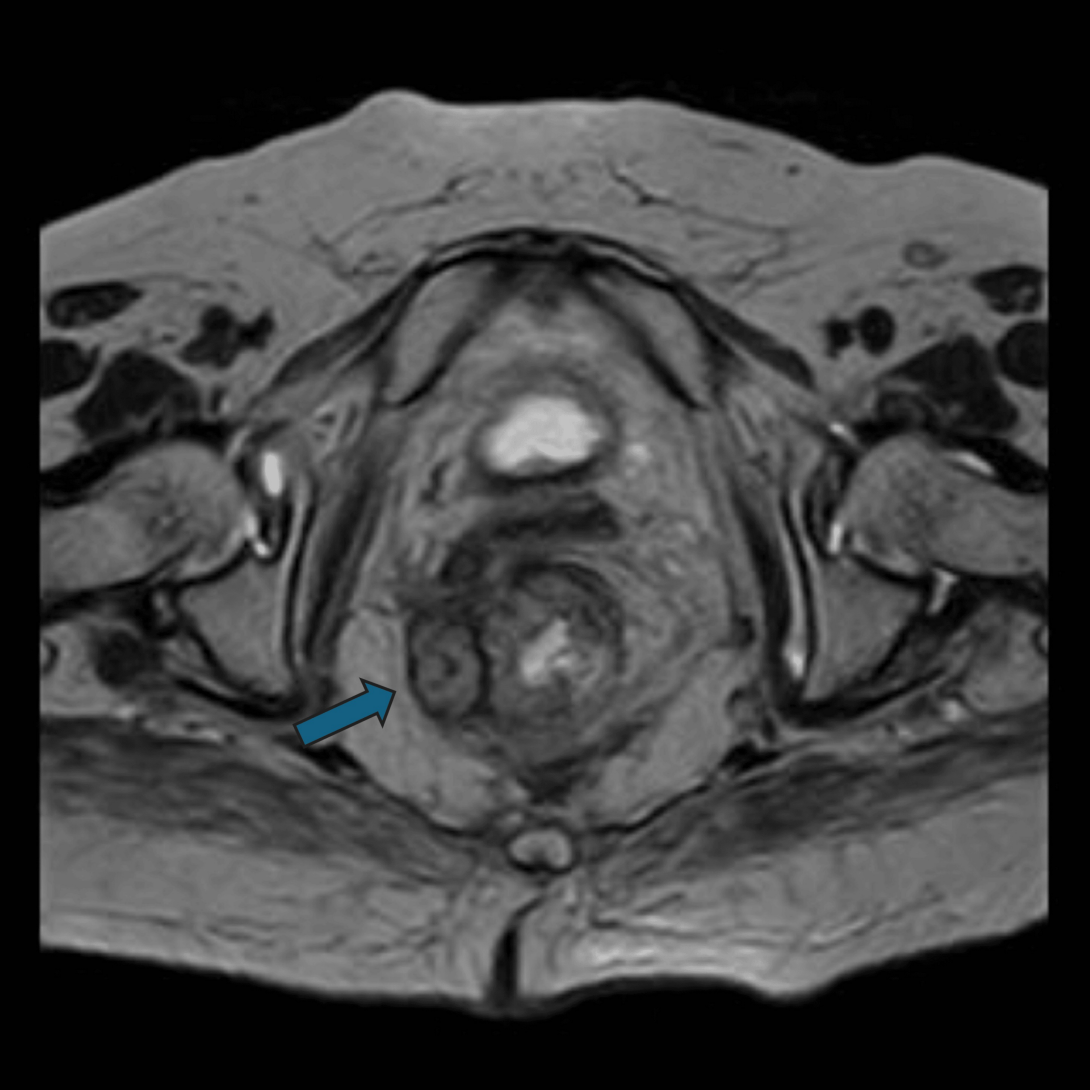
Magnetic resonance axial plane image T2 sequence showing reduction of the rectal lesion size after immunotherapy and before the abdominoperineal resection

One month later, the patient underwent external radiotherapy (total of 50 Gy), but the subsequent MRI did not reveal any improvement. She was then referred to our Surgical Center, and after consultation with the Medical Oncologist, we decided to start immunotherapy with nivolumab (anti-PD-1 monoclonal antibody) at a dosage of 480 mg every four weeks. After 12 treatment cycles, the patient underwent an MRI, which showed a significant size reduction of the rectal mass, measuring 3.2 × 3.3 × 3.9 cm (Figure 2).

Given the positive response to immunotherapy, and due to the previous open surgery and the size of the mass, an open approach with APR was decided. Under general anesthesia and in the lithotomy position, a midline incision was made. Despite the fact that only a loop sigmoidostomy was performed in the previous operation, multiple adhesions were found, mainly between the sigmoid colon and the lateral/anterior abdominal wall. Using electrocautery and LigaSure™ (Medtronic, Dublin, Ireland), the sigmoid colon was mobilized, and the colostomy was freed from the abdominal wall, with the opening sutured to avoid spillage. Central ligation of the vessels was performed. Complete mesorectal excision was done, and when the rectum was mobilized to the pelvic floor, the colon was divided, proximal to the previous stoma, using a linear stapler. Moving between the patient's legs and in the lithotomy position, an incision was made around the anal opening, and mobilization of the final part of the rectum was performed. The defect in the perineum was sutured with Vicryl and Prolene sutures (Ethicon, Somerville, NJ, USA). A 24F drain was placed in the pelvis. A new permanent colostomy was created, and after hemostasis, the abdominal wall was sutured. The postoperative course was uneventful, and the patient recovered well.

**Figure 3:**
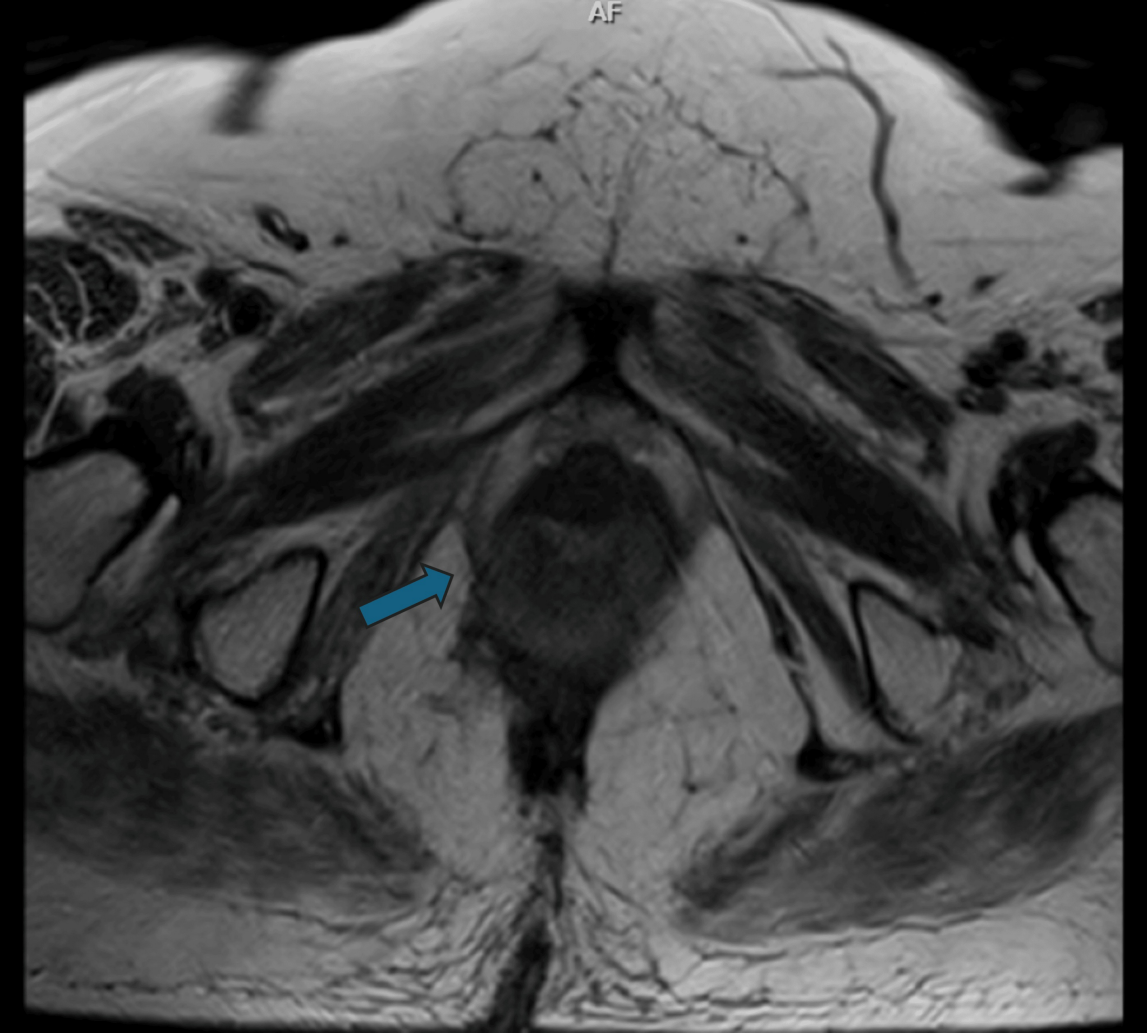
Magnetic resonance axial plane image T2 sequence showing complete response to surgery and long-term immunotherapy, without evidence of recurrence

During the following five years (until September 2024), the patient continued the same regimen of immunotherapy (nivolumab at a dosage of 480 mg every four weeks) without any reported adverse effects, in order to prevent any local recurrence and thereby improve the patient's survival. Currently, she is disease-free, with no evidence of recurrence on a five-year postoperative follow-up, as shown by the regular MRI (Figure 3).

## Discussion

All ARMs can be categorized by location as rectal (proximal to the dentate line), perianal (distal to the dentate line), or anorectal (infiltrating the dentate line). Regarding the staging system, tumors are classified as stage I if the disease is localized and lymph node-negative, stage II in cases of local disease with regional lymph node involvement, and stage III in cases of distant metastases [8]. The five-year survival rate is estimated to be 26.7% for stage I (median survival, 24 months), 9.8% for stage II (median survival, 17 months), and 0% for stage III (median survival, eight months) [8].

Since ARM is an aggressive mucosal tumor with an adverse prognosis, it is crucial to identify those clinicopathologic parameters that can affect disease-specific survival. The most significant risk factors associated with poor ARM outcomes are female sex, Caucasian race, advanced age, anal location of the tumor, stages II and III, involvement of more than one lymph node, and no chemotherapy [6,9]. Similarly, patients with tumor size <2 cm, absence of lymphovascular and perineural invasion, negative resection margin status, and superficial infiltration tend to have a favorable survival [10].

Considering the rarity of ARM and, therefore, the limited scientific data available, the optimal surgical treatment remains controversial. WLE is a conservative method with the purpose of achieving narrow negative resection (R0) margins. WLE has the advantages of low perioperative morbidity and preservation of anal function. On the other hand, APR is a more invasive procedure with en-bloc resection of the tumor and removal of the mesorectal lymph nodes. Therefore, APR may offer better locoregional control with lower recurrence rates, but it is associated with higher complication rates, the necessity of permanent colostomy, and a worse quality of life [11]. Recent studies showed that there was no significant difference in OS when comparing WLE versus APR, independent of tumor stage [3,5,12,13]. APR should be reserved for tumors that cannot be removed by WLE, for salvage surgery in case of recurrence, and if there is evidence of anal sphincter invasion or mesorectal lymph node infiltration [12].

A multimodal treatment approach, with the development of specific targeted therapies and immunotherapy, appears to be promising for patients with primary ARM. Given the lack of adequate published data on adjuvant therapies for ARM, most of the scientific evidence derives from studies published on immunotherapy for unresectable stages III and IV cutaneous and mucosal melanomas [7,14,15]. Nowadays, advancements in immune checkpoint inhibitors (ICIs) therapy (anti-CTLA-4 and anti-PD-1 monoclonal antibodies) have reduced tumor recurrence rates and, therefore, improved OS in patients with ARM [16-18]. The ICI can be administered as monotherapy or in combination, and in neoadjuvant and/or adjuvant settings in patients with ARM. Ogata et al. reported that the two-year OS was significantly improved in patients with metastatic ARM who received ICI therapy compared to those who received dacarbazine (median survival, 28.6 months vs. 16.0 months; two-year OS rate, 61.4% vs. 0%; p = 0.048) [16], while Ho et al. reported a three-year OS rate of 55% in patients who received neoadjuvant immunotherapy with ICI for resectable mucosal melanoma [17]. Taylor et al. reported that ARM patients who received immunotherapy had significantly better two-year OS rates than those who did not receive immunotherapy (49.21% vs. 42.47%, p = 0.03), with an increased immunotherapy utilization over the years (7.24% in 2004, 21.27% in 2015, p < 0.001), where younger age, positive nodes, and larger tumor size were identified as independent predictors of immunotherapy treatment [4].

In our report, the patient initially received neoadjuvant ICI therapy with nivolumab (anti-PD-1 monoclonal antibody), demonstrating a positive response with a reduction in tumor size sufficient for a successful APR. Thereafter, with the surgical approach, we achieved complete tumor resection with clear margins, and the patient continued long-term immunotherapy with the same adjuvant ICI regimen as preoperatively, remaining disease-free at a five-year follow-up after surgery. We emphasize the necessity of combined therapeutic strategies to offer durable locoregional disease control, and therefore, to improve survival in patients with early-stage ARM.

## Conclusions

Patients with ARM have a poor prognosis due to an aggressive tumor biology and the non-specific initial presentation, making early diagnosis challenging. Complete surgical resection with negative margins is the first-line treatment, and, if combined with long-term neoadjuvant-adjuvant immunotherapy, may improve outcomes in ARM patients. Our case emphasizes the importance of a multidisciplinary approach with experts in ARM surgery and systemic therapies to offer a better prognosis and, therefore, guarantee a good quality of life in patients with ARM. Further scientific research is needed to develop effective treatment strategies for this rare disease.
